# Determination of patulin in products containing dried fruits by Enzyme‐Linked Immunosorbent Assay technique Patulin in dried fruits

**DOI:** 10.1002/fsn3.2386

**Published:** 2021-06-21

**Authors:** Anna Przybylska, Agnieszka Chrustek, Dorota Olszewska‐Słonina, Marcin Koba, Stefan Kruszewski

**Affiliations:** ^1^ Department of Toxicology and Bromatology Faculty of Pharmacy L. Rydygier Collegium Medicum in Bydgoszcz Nicolaus Copernicus University in Torun Bydgoszcz Poland; ^2^ Department of Pathobiochemistry and Clinical Chemistry Faculty of Pharmacy L. Rydygier Collegium Medicum in Bydgoszcz Nicolaus Copernicus University in Torun Bydgoszcz Poland; ^3^ Medical Physics Division Biophysics Department Faculty of Pharmacy L. Rydygier Collegium Medicum in Bydgoszcz Nicolaus Copernicus University in Torun Bydgoszcz Poland

**Keywords:** dried fruits, ELISA, herbal blends, patulin, rowan fruit, tea

## Abstract

The era of globalization causes that the export and import of food from different continents of the world are becoming more and more common, which may directly contribute to the increase in pollution in them. The presence of mycotoxin in food is an ubiquitous problem. There is very limited information on the possible influence of the composition of herbal mixtures on the presence of mycotoxins in them, which is an area where research can be expanded. The aim of this study was to determine patulin (PAT) in commercial products containing dried elderberry, rose, blueberry, rowan, hawthorn, and chokeberry fruits by enzyme‐linked immunosorbent assay technique. Research using this technique allowed for considering the possible influence of the composition of herbal mixtures on the concentration of patulin in them. Patulin was detected in all analyzed samples with wide range of <LOD ÷ 4,102.0 µg/kg. In 91% of the single‐ingredient products, the mean patulin concentration below 50 µg/kg was found. We observed that patulin content in products containing only rose, elderberry, blueberry, rowan, hawthorn, or chokeberry fruit was lower than in herbal blends. Research showed that adding dried rowan fruits to herbal blends may contribute to a decrease in PAT levels (r = 0.8581; *p* = .0031). Looking for such technological methods creating the most unfavorable conditions for the biosynthesis of patulin in medicinal raw materials is extremely important from the point of view of medical and pharmaceutical care.

## Graphical abstract

The conducted research indicates the possibly of using the ELISA method for the determination of PAT in materials of plant origin. Research using this technique allowed for considering the possible influence of the composition of herbal mixtures on the concentration of patulin in them. Research showed that adding dried rowan fruits to herbal blends may contributes to a decrease in PAT levels (r = 0.8581; *p* = .0031).

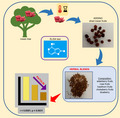

## 1 INTRODUCTION

Poland is a country with a strong tradition of using raw materials of plant origin for medicinal purposes. The transfer of knowledge about the benefits of using plant materials is very often passed on from generation to generation. In the world market, as much as 80% of plant raw materials come from wild cultivation (Asl Roosta et al., 2017). The era of globalization causes that access to food from different parts of the world is not a problem. Almost 90% of apples and raw ingredients for apple products are imported in Taiwan (Lien et al., 2020). In turn, in Afghanistan, raisins are the main export of all agricultural products. In this country, the value of the export market is estimated at 17% (McCoy et al., 2015). The export and import of food from different continents of the world is becoming more and more common, which may directly contribute to the increase in pollution in them (Fritsche, 2018; Kendall et al., 2018). A literature review shows that the presence of mycotoxin in food is an absolutely ubiquitously problem (Moretti et al., 2017). The presence of mycotoxins in food is associated with a twofold problem. The first is economic losses, as it turns out that up to 25% of food is exposed to mycotoxins, which directly translates into losses in agriculture (Moretti et al., 2017). The second, extremely important problem, is the immunotoxic, neurotoxic, and dermotoxic effect of patulin (PAT) on animals and human (Pal et al., 2017; Przybylska et al., 2019). In experimental animals, patulin causes hemorrhages, formation of edema, and dilation of the intestinal tract. In addition, in some cases it causes convulsions, dyspnea, pulmonary congestion, edema, ulceration, hyperemia, and distension of the gastrointestinal tract (Pal et al., 2017). Patulin also poses a significant threat to humans, causing allergic reactions (Sohrabi et al., 2021). Thus, the search for natural, eco‐friendly ways to reduce the amount of patulin in plant raw materials seems to be extremely important from the economic and health point of view.

Patulin (4‐hydroxy‐4H‐furo(3,2c)pyran‐2 (6H)‐one) is a stable in aqueous media at 105–125℃ with melting point of 110℃ (Hussain et al., 2020). This mycotoxin is produced by approximately 60 species, such as *Penicillium, Aspergillus, Byssochlamys, Alternaria,* and *Paecilomyces,* belonging to >30 genera of fungi (Drusch & Ragab, 2003; Pal et al., 2017; Przybylska et al., 2019). PAT was isolated in 1943 from *Penicillium griseovulum* mold by Birkinshaw et al. (Puel et al., 2010) and initially was valued for its antibiotic properties against gram‐positive and gram‐negative bacteria (Ioi et al., 2017). In addition to the digestive tract, PAT can enter the body through the respiratory tract, for example by inhaling dust. Despite the low concentration of mycotoxins in dust, their absorption by inhalation may contribute to an increased risk of poisoning not only with patulin, but also with ochratoxin A, deoxynivalenol, and zearalenone (Tangni & Pussemier, 2007).

Due to its properties of patulin, the World Health Organization (WHO) recommends limiting the maximum PAT content in apple to 50 µg/kg, in apple puree 25 µg/kg, and in baby food to 10 µg/kg (Hussain et al., 2020). There are no directives relating to the presence of patulin in medicinal plant. The Joint Expert Committee on food Additives of the World Health Organization (JECFA) recommended that daily human exposure to PAT should be reduced to 0.4 µg/kg body weight per day (Hussain et al., 2020; Przybylska et al., 2019). The major source of PAT in food are apples, pears, mango fruits, orange fruits, cherries, grapes, and fruit juices (Hussain et al., 2020; Pal et al., 2017). It turns out that PAT is also present in the products containing hawthorn berries (Ji et al., 2017; Li et al., 2007; Przybylska et al., 2019; Xiang et al., 2012; Zhou et al., 2012). The literature review shows that there are still no studies on the assessment of patulin content in commercial plant raw materials and herbal blends containing dried elderberry, rowan, chokeberry, blueberry, or rose fruits.

Taking into account the above considerations, the aim of this study was to determine PAT in dried herbal products containing only one dried component and herbal blends with dried different parts of medicinal plant. In addition, the studies attempted for the first time to determine the effect of the presence of individual components of the herbal blend on the final concentration of PAT in them.

## 2 MATERIALS AND METHODS

Thirty‐one commercial products were analyzed in this study (See Tables 1 and 2). All of them were purchased from supermarkets, herbal stores, and pharmacies in Bydgoszcz (Poland) and by internet sale in January 2020. All the samples (*n* = 31) were grouped into products single‐component commercial products (SC; *n* = 22) and multicomponent commercial herbal blends containing different dried parts of various medicinal plants (MC; *n* = 9). Single‐component (SC) and multicomponent (MC) commercial herbs were packed in collective packages or in sachets. Bags were selected at random for each packaged product, and bulk samples were taken from three different parts of the pack. These products contained dried parts of various medicinal plants belong to eight different families (*Rosaceae*, *Adoxaceae*, *Ericaceae*, *Grossulariaceae*, *Berberidaceae*, *Malvaceae*, *Polygonaceae*, *Scabiosa*) where three of them originated from organic farms but five of them were dietary supplements. All analyzed samples were stored in their original packaging until analysis. During the research, the use‐by date of the analyzed products was taken into account. All the samples were analyzed at least twice.

**TABLE 1:** Characteristic data of studied types of analyzed single‐component commercial herbal blends (SC)

	Symbol of sample	Producer	Composition (100%)	Family^d^	Kind of package	Comments^b^
1	P35	A1	rose fruit	*Rosaceae*	CP ^a^ (50 g)	‐
2	P40	A2	rose fruit	*Rosaceae*	CP^a^ (50 g)	‐
3	P33	A1	rose fruit	*Rosaceae*	CP^a^ (50 g)	‐
4	P38	A1	rose fruit	*Rosaceae*	30 × 2.0 g	‐
5	P36	A3	rose fruit	*Rosaceae*	CP^a^ (50 g)	>300 mg/100g ascorbic acid
6	P39	A2	rose fruit	*Rosaceae*	CP^a^ (50 g)	organic farmic producer
7	P37	A3	rose fruit	*Rosaceae*	CP^a^ (50 g)	dietary supplements
8	P32	A1	rose fruit	*Rosaceae*	CP^a^ (50 g)	‐
9	P13	A4	elderberry fruit	*Adoxaceae*	CP^a^ (100 g)	origin: Poland
10	P14	A1	elderberry fruit	*Adoxaceae*	CP^a^ (50 g)	‐
11	P16	A1	elderberry fruit	*Adoxaceae*	CP^a^ (50 g)	‐
12	P17	A2	elderberry fruit	*Adoxaceae*	CP^a^ (100 g)	organic farmic producer
13	P11	A3	blueberry fruit	*Ericaceae*	CP^a^ (50 g)	‐
14	P10	A5	blueberry fruit	*Ericaceae*	CP^a^ (50 g)	OTC^c^
15	P18	A6	rowan fruit	*Rosaceae*	CP^a^ (50 g)	origin: Poland
16	P19	A3	rowan fruit	*Rosaceae*	CP^a^ (50 g)	dietary supplements
17	P20	A3	rowan fruit	*Rosaceae*	CP^a^ (50 g)	dietary supplements
18	P3	A3	hawthorn fruits	*Rosaceae*	CP^a^ (50 g)	dietary supplements
19	P5	A1	chokeberry fruit	*Rosaceae*	CP^a^ (50 g)	‐
20	P8	A1	chokeberry fruit	*Rosaceae*	CP^a^ (50 g)	‐
21	P9	A1	chokeberry fruit	*Rosaceae*	CP^a^ (50 g)	‐
22	P7	A6	chokeberry fruit	*Rosaceae*	CP^a^ (100 g)	origin: Poland

^a^ CP ‐ collective packaging.

^b^ Manufacturer's declaration.

^c^ OTC ‐ Over‐the‐counter drug.

^d^ Family of the fruit underlined in the next column in table.

^×^ Not defined content.

**TABLE 2:** Characteristic data of studied types of analyzed multicomponent commercial herbal blends (MC)

	Symbol of sample	Pr*	Composition	Family^d^	Kind of package	Comments^b^
1	P22	A7	hibiscus (41%), apple (15,5%), rose fruit (15%), blackcurrant fruit (10%), aromas^×^, chokeberry fruit (5%), elderberry fruit (2%), blackberry (1%), blackberry leaf, raspberry (1%), strawberry (1%)	*Malvaceae*	20 × 2.0 g	‐
2	P25	A2	rose fruit^×^, sloe fruit^×^, apples^×^, barberry fruit^×^, elderberry fruit^×^, rowan fruit^×^, blackcurrant fruit^×^, raspberry^×^, hawthorn fruit^×^, hibiscus^×^, rose petals^×^	*Rosaceae*	CP^a^ (100 g)	organic farmic producer
3	P30	A8	chokeberry fruit (20%), hibiscus (16%), hawthorn fruit (16%), hawthorn inflorecences (12%), rowan fruit (12%), black tea (10%), buckwheat hulls (4%), valerian root (4%), elderberry fruit (2%), blackcurrant fruit (2%), cherry fruit (2%)	*Rosaceae*	60 × 2.5 g	dietary supplements
4	P27	A9	elderberry fruit^×^, fruit berries (15%), chokeberry fruit (14%), apples (13%), hawthorn fruit (10%), hawthorn inflorescences (10%), rosehip skin^×^, rowan fruit (5%), aromas^×^	*Adoxaceae*	CP^a^ (100 g)	‐
5	P23	A10	hibiscus petal^×^, rowan fruit^×^, sloe fruit^×^, hawthorn fruit^×^, raspberry fruit^×^, elderberry fruit^×^, chokeberry fruit^×^	*Malvaceae*	CP^a^ (50 g)	‐
6	P31	A5	rose fruit (66%), hibiscus^×^, elderberry fruit^×^, sloe fruit^×^	*Rosaceae*	20 × 3.5 g	‐
7	PX38	A5	chokeberry fruit (55%), hibiscus^×^, hawthorn fruit^×^, rose fruit^×^, blackberry leves^×^, aromas^×^	*Rosaceae*	20 × 3.5 g	‐
8	P21	A9	elderberry fruit (55%), chokeberry fruit^×^, hibiscus^×^, apple^×^, aronia juice concentrate^×^, citric acid^×^	*Adoxaceae*	25 × 2.0 g	‐
9	P29	A11	hibiscus^×^, apple^×^, rose fruit^×^, blackberry leaves^×^, lemon flavor^×^, blackcurrant flavor^×^, elderberry fruit^×^, blackcurrant fruit (1%), lemon peel (1%)	*Malvaceae*	20 × 2.5 g	origin: EU

^a^ CP ‐ collective packaging.

^b^ Manufacturer's declaration; EU ‐ European Union.

^d^ Family of the fruit underlined in the next column in table.

^×^ Not defined content.

* Pr ‐ producer.

Most of the reagents used in this study were contained in the Patulin ELISA Test Kit, which included microtiter plate with patulin standards, patulin‐HRP conjugate, extraction buffer, wash solution, stop buffer, and TMB substrate. Methanol for HPLC was obtained from POCH (Poland).

To analyze the PAT content in dried samples, commercial Patulin ELISA Test Kit (Reagen^TM^, USA) was used. All steps of the analysis were carried out in accordance with the manufacturer's assay procedure. Four grams of samples was added to 20.0 ml methanol for HPLC (POCH, Poland), and the solution was shaken for 400 rpm. Then, 8.0 ml of the homogenized sample was centrifuged for 5 min at 3,500 × g for 5 min at room temperature (22℃). After that, extract was filtered with use of a filter paper and a Chromafil PES‐45/25 syringe filter (Mecherey‐Nagel, Germany). The supernatant (0.5 ml) was transferred to a tube and added 0.5 ml Sample Extraction Buffer. The mixture was mixed before analysis. One hundred µL of the patulin standard (0.0 ng/ml ÷ 1,000.0 ng/ml) and test samples (100.0 µl/well) was added to the wells of microtiter plate and added 50.0 µl of patulin‐HRP Conjugate (patulin horseradish peroxidase) to each well and mix well by gently rocking the plate manually for 60 s. Then, the plate was incubated for 60 min at room temperature (22℃). After the washing step (3 × 250.0 µl), 100.0 µl of the enzyme conjugate was added and incubated for 20 min at room temperature (22℃) in the dark. Following the addition of 100.0 µl of the stop reagent to each well, the absorbance was measured at 450 nm in ELISA reader (Thermo Scientific, Finland).

The bulk density (BD) of the tested samples was determined according to the method of Ogrodowska et al. (2011).

**FIGURE 1:**
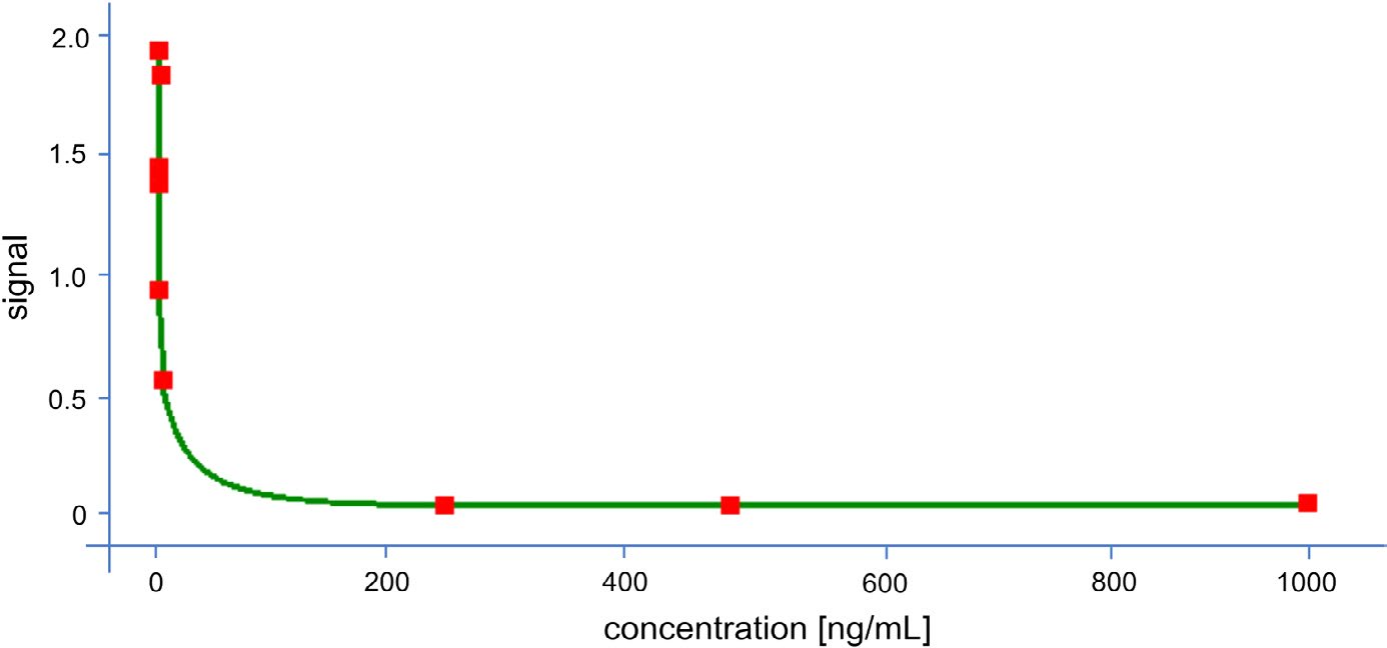
Calibration curve of patulin standard

According to the REAGEN Patulin ELISA Test Kit guidelines, the recovery rate of patulin is between 75% and 95% and the specificity (cross‐reactivity) is 100%. The limits of detection (LOD) for patulin were 0.1 ng/ml. Nine standard solution of patulin

(0,0 to 1,000,0 ng/ml) have been used for the calibration curve (R2 = 0.994) (See Figure 1). The equation of the trendline was shown below:

$$y=1,94+\frac{0,04‐1,94}{1+{\left(\frac{x}{1,44}\right)}^{‐0,79}}$$

### 2.1 Statistical analysis

The obtained results were analyzed statistically with Statistica v.12 (StatSoft, USA). The results (duplicate samples) are presented as the mean of PAT content in analyzed products. In turn, the results of PAT concentration in the analyzed products groups were shown as the median and quartile range. The Shapiro–Wilk test was used for each data sets. The *p*‐value <.05 was considered significant. To evaluate the difference between samples, a nonparametric Mann–Whitney U test was used (*p* < .05). We also used the Spearman correlation to determine relationships between the relevant variables and the concentration of patulin.

## 3 RESULTS AND DISCUSSION

Since 1970, the popularity of the use of immunoenzymatic methods has increased in the analysis of mycotoxins (Singh & Mehta, 2020). In recent years, ELISA test kits are used to determine concentration of mycotoxins such as patulin (PAT), aflatoxins (AFs, AFB_1_), ochratoxins (OTA), zearalanone (ZEN), trichothecenes, fumonisins (FBs), citrinin (CIT) in food samples, and agricultural commodities (Azam et al., 2021; Omar et al., 2020; Puga‐Torres et al., 2020; Sudakin & Fallah, 2008; Tang et al., 2019). The ELISA technique has also been used in herbal raw materials such as black pepper, coriander, ginger, garlic, and plants belonging to the *Fabaceae* family also (Dungkokkruad et al., 2017; Orina et al., 2020; Thirumala‐Devi et al., 2001; Tonti et al., 2017; L. Zhang et al., 2018). The applied ELISA method for the determination of PAT in plant dried materials was described for the first time. It should be noted, however, that although ELISA technique give quick and economical measurements, they lack precision at low concentration (Singh & Mehta, 2020).

In own research, the PAT content in the analyzed commercial single‐component (SC) and multicomponent (MC) products is shown in Table 3. The highest average PAT content (4,102.0 µg/kg) was identified in sample number P29 containing hibiscus, apple, rose fruit, blackberry leaves, lemon flavor, blackcurrant flavor, elderberry fruit, blackcurrant fruit (1%), and lemon peel (1%) made from European Union. According to the manufacturer's declaration, this product was produced in European Union. In turn, the concentration of PAT below limit of detection was determined in dried 100% hawthorn fruits (P3) and rose fruit (P35) that comes from Poland.

**TABLE 3:** Natural occurence of PAT in analyzed products

	Percentage of PAT positive samples	Range of PAT content [µg/kg]	Median of PAT content [µg/kg]	Q_1_ *	Q_3_ *	R_q_ *
Single‐component commercial products (*n* = 22)	20/22 (91%)	<LOD ÷268.3	5.0^A^	2.4	12.8	10.4
Multicomponent commercial products (*n* = 9)	9/9 (100%)	5.8 ÷ 4,102.0	63.4^A^	39.4	774.5	735.1

* – Q_1_ – lower quartile, Q_3_ – upper quartile, R_q_ – quartile range; within SC and MC groups means signed by different capital letters differ at *p* < .05.

We observed that PAT content in SC products containing only rose fruit, elderberry fruit, blueberry fruit, rowan fruit, hawthorn fruit, or chokeberry fruit was significantly different (*p* < .05) and lower than in samples with the mixture of dried herbal materials (MC). Despite the highest concentration of PAT in SC products of 268.3 µg/kg for dried rowan fruits (Table 4), 91% (20/22) of SC products had a content of this mycotoxin below 50 µg/kg. Considering herbal raw materials containing only one ingredient (SC), it was found that the highest PAT content was recorded for dried rowan fruit (25.7 µg/kg), but the lowest was recorded for dried hawthorn fruits (<LOD). These results were presented in Table 4. After the analysis, it was found that the median of PAT content in rowan fruits (SC) is almost three times higher than in the blueberry and chokeberry fruit and almost 10 times higher than rose fruit. Five products (Tables 1, 2), defined by the manufacturer as a “dietary supplement” were used in the research. It was found that the concentration of PAT in dietary supplements reached an average of 67.5 µg/kg. In addition, current studies have reported a similar median concentration of PAT in dried fruits (SC) belonging to the *Adoxaceae* and *Rosaceae* family (Table 5). During the analysis, it was found that in fruits belonging to the *Ericaceae* (blueberry fruits), the median of PAT was over two times lower than in fruits belonging to *Rosaceae* and *Adoxaceae*.

**TABLE 4:** Natural occurence of PAT in analyzed dried fruits (SC products)

Family	Dried fruit	Symbol of samples	Range of PAT content [µg/kg]	Median of PAT content [µg/kg]	Q_1_ *	Q_3_ *	R_q_ *
*Adoxaceae*	elderberry fruit (*n* = 4)	P13, P14, P16, P17	2.1 ÷ 79.0	7.9	3.2	45.3	42.1
*Ericaceae*	blueberry fruit (*n* = 2)	P10, P11	5.7 ÷ 14.0	9.9	5.7	14.0	8.3
*Rosaceae*	rowan fruit (*n* = 3)	P18, P19, P20	3.6 ÷ 268.3	25.7	3.6	268.3	264.7
	hawthorn fruit (*n* = 1)	P3	<LOD	<LOD	<LOD	<LOD	<LOD
	rose fruit (*n* = 8)	P35, P40, P33, P38, P36, P39, P37, P32	0.3 ÷ 6.0	2.8	0.8	3.5	2.8
	chokeberry fruit (*n* = 4)	P5, P7, P8, P9	6.8 ÷ 14.9	10.5	7.5	13.8	6.3

* – Q_1_ – lower quartile, Q_3_ – upper quartile, R_q_ – quartile range.

**TABLE 5:** Natural occurence of PAT in fruits (SC products) belongs to *Adoxaceae*, *Ericaceae,* and *Rosaceae*

Family	Symbol of samples	Range of PAT content [µg/kg]	Median of PAT content [µg/kg]	Q_1_ *	Q_3_ *	R_q_ *
*Adoxaceae* (*n* = 4)	P13, P14, P16, P17,	2.1 ÷ 79.0	7.9	3.2	45.3	42.1
*Ericaceae* (*n* = 2)	P10, P11	5.7 ÷ 14.0	9.9	5.7	14.0	8.3
*Rosaceae* (*n* = 16)	P35, P40, P33,P38, P36, P39, P37, P32, P18, P19, P20, P3, P5, P8, P9, P7	<LOD ÷268.3	3.6	1.7	10.5	8.8

* – Q_1_ – lower quartile, Q_3_ – upper quartile, R_q_ – quartile range.

**FIGURE 2:**
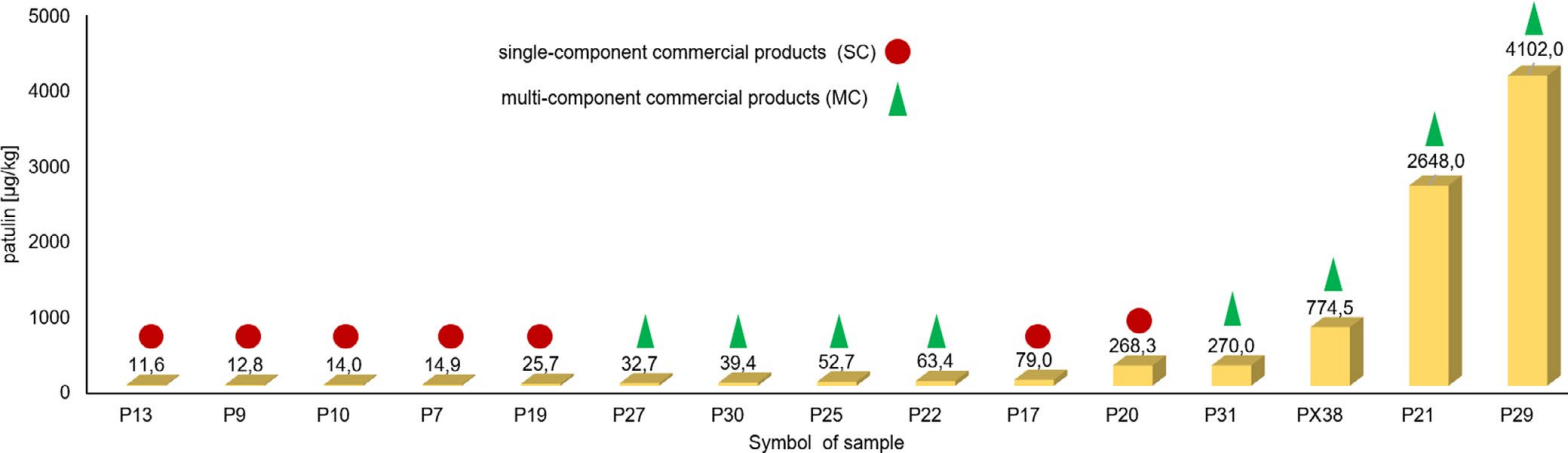
Comparison of average of PAT in the analyzed samples where concentration of patulin was higher than the maximum tolerance limit of 10 µg/kg recommended by WHO for baby food (Hussain et al., 2020)

All the multicomponent (MC) products used in the research were a mixture of dried fruit, flowers and leaves (Table 2). All the analyzed MC samples were contaminated by PAT at the wide range of 5.8 ÷ 4,102.0 µg/kg what was shown in Table 3. The obtained results show that herbal blends containing additional intermediates in the form of aromas and/or citric acid (PX38, P21, and P29) characterized by a higher concentration of PAT than products consisting only of natural ingredients—dried fruits, leaves, hulls, and root. See Figure 2. During the analysis of the results, it was found that samples eleven‐components P22 (63.4 µg/kg), P25 (52.7 µg/kg), and P30 (39.4 µg/kg) contained a lower average concentration of PAT compared with four‐component P31 (270.0 µg/kg), six‐component PX38 (774.5 µg/kg), and P21 (2,648.0 µg/kg). This relationship is confirmed by a positive correlation between the amount of ingredients in a given products (SC and MC) and the concentration of PAT in them (r = 0.6169; *p* < .0002). Correlation Spearman's also showed positive significant relationship between presence of dried rowan fruit in MC products and concentration of PAT in final herbal blends (r = 0.8581; *p* = .0031). We observed that average PAT content in MC products containing dried rowan fruit was significantly different (*p* < .05) and lower compared to products without it (P31, PX38, P21, P29). In the samples P25, P30, P27, and P23, in which producers declared the presence of rowan fruit, the average PAT concentration was, respectively: 52.7; 39.4; 32.7, and 5.8 µg/kg. Moreover, correlation analysis (Spearman’ correlation) allowed to note a monotonic direct relationship between the concentration of PAT in dried multi‐component products and their degree of fragmentation expressed as g/mL (r = 0.5833; *p* = .0992). The range of bulk density for SC and MC products was determined between 0.23 ÷ 0.56 and 0.17 ÷ 0.58 g/ml. In current studies, we also found that the content of PAT in all analyzed products depends on the method of packing plant raw materials, because the concentration of this mycotoxin is higher for products packed in sachets than in collective packaging (r = 0.6628; *p* < .0005).

In addition, analytical results suggested that only in 26% of the analyzed dried commercial products concentration of PAT was higher than the maximum tolerance limit of 50 µg/kg recommended by WHO (Drusch & Ragab, 2003; Przybylska et al., 2019). See Figure 2. Moreover, in the 11 samples (36%) PAT content was found exceeded the WHO acceptable upper limit of 25 µg/kg that is recommended for apple puree. Also, our results showed that in the 15 analyzed samples (48%), the concentration of PAT was higher than 10 µg/kg, which is the tolerance upper limit indicated by WHO for baby food. In 5 samples of 31 (16%), an average concentration of PAT above 100 µg/kg was recorded (Figure 2).

The current studies have shown that lower concentration of PAT has been reported for products containing only one dried ingredients (SC) compared with multicomponent products (MC), which is confirmed by previous studies using ultra‐high performance liquid chromatography coupled with tandem mass spectrometry (UHPLC‐MS/MS) method (Przybylska et al., 2019). In the cited research, it was found a lower average concentration of PAT for dietary supplements (11.4 ± 5.5 µg/kg) formulated with hawthorn fruits from *Crataegus monogyna/Crataegus laevigata* trees compared with multicomponents products containing mostly dried hawthorn fruits (15.7 ± 31.6 µg/kg). Additionally in cited paper, PAT was not detected in the frozen hawthorn fruits (*Crataegus monogyna*) mature hawthorn trees located in Fordon and Bartodzieje district in Bydgoszcz (Przybylska et al., 2019). On the other hand, in previous studies, dried hawthorn fruits were obtained in the product from organic farming (Poland), and the average PAT concentration was 9.1 ± 1.1 μg/kg (Przybylska et al., 2018). In the current study, the lowest PAT concentration was recorded in dried hawthorn fruits and rose fruit (P3, P35; < LOD µg/kg). Compared with the work of Ji et al. (2017), PAT in dried hawthorn products was determined in 10% of all analyzed samples with a mean content of 11.1 µg/kg. Studies by the other authors have shown that packaged midland hawthorn berry (*Crataegus laevigata*) tea was the source of eight mycotoxins: deoxynivalenol (2,874 µg/kg), T‐2 (60.3 µg/kg), HT‐2 (12.3 µg/kg), four enniatins (2.44 ÷ 11.70 µg/kg), and beauvericin (4.5 µg/kg) (Reinholds et al., 2019). However, it should be noted that the studies did not include the assay in the PAT samples analyzed. Extensive research in Lithuania shows that 45% of "teas" samples of various species shows the presence of deoxynivalenol at levels between 129 and 5,463 µg/kg. Moreover, six "teas" containing rose fruits (dog fruits), midland hawthorn fruits, St. John‘s Wort, purple coneflower, and herbal blend (mixture of birch, bearberry, knotgrass, rest harrow, parsley, nettle, yarrow, elderberry) contained deoxynivalenol over 2000 µg/kg (Reinholds et al., 2020).

In our own research, in dried elderberry fruits (*Vaccinium myrtillus*) PAT was detected at a range of 2.1 ÷ 79.0 µg/kg (Table 5). For comparison, in the fresh highbush blueberry (*Vaccinium corymbosum*) that were grown in the Republic of Belarus (Zenkova & Pinchykova, 2019) and in raspberries, blueberries, blackberries, and such cherries from Czech Republic patulin was not found (Vaclavikova et al., 2015). Similar results obtained for 31 samples of dried organic cranberries and two samples of organic bilberry press cake (Popa et al., 2019). Popa et al. (2019) using high‐performance liquid chromatography with diode array detection (HPLC‐DAD) method in purified and concentrated extracts of the samples which were received in lyophilized form, PAT also was not detected. In turn, research Drush & Ragab (2003) showed that raspberries can be a source of PAT with a concentration of up to 746 µg/kg, which may be an indirect reason for the presence of PAT in herbal blends P22 (63.4 µg/kg), P25 (52.7 µg/kg), and P23 (5.8 µg/kg).

Very high PAT concentrations have been reported in the herbal blends containing different dried parts of medicinal herb, such as rose fruit (dog fruits), chokeberry fruit, hawthorn fruit, apples, and hibiscus flower (Table 3). The highest average content of PAT obtained for P29 sample (4,102.0 µg/kg). Likewise, a high concentration of PAT was described by Zhang et al. (2016). Authors of this paper determined the concentration of PAT in 60% (9/15) of raw samples of Pu‐erh tea by LC‐MS/MS at an average level of 1,169 µg/kg, while in 12.5% of ripened samples (2/16) at an average level of 915 µg/kg. While *Aspergillus* and *Penicillium* are considered the most important from a food safety (Amoah et al., 2020), the authors identified *Aspergillus niger,* but patulin‐forming fungi such as *Penicillium expansum, Penicillium griseovulum, Penicillium carneum, Penicillium glandicola, Penicillium coprobium, Penicillium vulpinum, Penicillium, clavigenum, Penicillium concentricum*, *Paecilomyces variotti,* and *Byssochlamys nivea*—were not determined (Zhang et al., 2016). Research shows that *Aspergillus* contamination of plant‐based raw materials (garlic) comes from the field and persists through processing, including washing and drying (Amoah et al., 2020).

The presence of patulin‐producing species of fungi is not the only condition that determines the increase in PAT biosynthesis. The biosynthesis of mycotoxins, including PAT, depends on many different physicochemical parameters. Some of them are water activity, and the temperature maintained during the storage of food products and their pH (Tannous et al., 2016). The intensity of PAT biosynthesis also depends on variety of fruits used in the production process, content of organic acids leached from the vacuole and to the mycelium of *P. expansum,* as well as abiotic factors such as humidity, temperature, and sunlight (Barad et al., 2016; Drusch & Ragab, 2003). The wide range of PAT concentration in the tested products may result from the use of different apple varieties, as the research of Barad et al. (2016) where suggested that cultivars of apple trees are an important factor influencing the inhibition of PAT biosynthesis. Nevertheless, noncontrolled use of apple leather to the herbal blends can be additional and significant source of PAT in products. The research of Montaseri et al. (2013) showed that apple leather can be infected with PAT in a wide range, up to <10 ÷ 2,559 µg/kg. By analyzing content of PAT in MC commercial herbal blends, we claimed that, according to the manufacturer's declaration, the sample P23 containing the most of hibiscus petals and dried rowan fruits characterized the lowest average concentration of PAT – 5.80 µg/kg. Similar results were obtained by Przybylska et al. (2019). In this study, in the sample where the largest share, according to the producer's declaration, was hawthorn fruit, lemon balm leaf, and hibiscus flower, the concentration of PAT was significantly lower compared with products containing more dried fruits than leaves. It should be added that the presence of deoxynivalenol, aflatoxin B1, HT‐2, T‐2, zearalenone, sterigmatocystin, ochratoxin, enniatins, and beauvericin was not found in the bearberry leaves, eucalyptus leaves, linden flowers, calendula, yarrow, and yellow everlasting flower tea (Reinholds et al., 2019), which are a rich source of biologically active compounds. In recent years, attention has also been paid to the unusual properties of propolis. Studies have shown that 2 mg/ml propolis extract reduces the growth of PAT in apples juices (Silici & Karaman, 2014). Propolis is collected by honey bees, making it a rich source of aromatic acids, aromatic esters, volatile compounds, aromatic compounds, hydrocarbons, steroids, flavonoids, acids, micro‐ and macronutrients, vitamins, and essential oils (Hemmami et al., 2020; Pobiega et al., 2019). The abundance of bioactive compounds present in propolis or cinnamon oil which could reduce the expression of genes involved in PAT biosynthesis (Lai et al., 2021) suggests that the composition of herbal blends may also play a role in controlling optimal PAT growth conditions. The exogenous amino acid L‐glutamate has strong properties that inhibit the development of blue rot caused by *Penicillium expansum* in the postharvest pear fruit (Jin et al., 2019). Literature data confirm that *Camelia sinensis* is source of γ‐aminobutyric acid (Przybylska et al., 2021), but rowan fruits (*Sorbus aucuparia* L.) are richer source of amino acids than hawthorn fruits (*Crataegus sanguinea* Pall.) or cinnamon fruits (*Rosa cinnamomea* L.) (Sergunova et al., 2020). In turn, hibiscus (*Hibiscus* L.), rowan fruits, blackcurrant fruits (*Ribes nigrum*), or raspberry (*Rubus idaeus*) are richer source of amino acids compared with apples (*Malus domestica*) or redcurrant (*Ribes rubrum*) (Kunachowicz et al., 2018; Sergunova et al., 2020). In current study, the authors found the highest average PAT content in dried rowan fruit. See Table 4. Interestingly, at the same time, the lowest PAT concentration was recorded in multi‐ingredient products with the addition of dried rowan. The authors speculate that it may be caused not only by the presence of amino acids in the dried fruit, but also by competition from microorganisms in the product. It should also be noted that in the P29 product, in which the presence of PAT above 4,000 µg/kg was found, there was no dried rowan fruit. Interaction between mycotoxin and selected herbs and species components was described by Do et al. (2015). One of the most important mechanisms in biocontrol is competition for the living space and nutrients of numerous yeasts and molds, what may explain the significant decrease in PAT in herbal blends with dried rowan fruit (Spadaro et al., 2013). Numerous studies prove PAT degradation using a biocontrol mechanism. For example, strain of *Metschnikowia fructicola* AL27 is more effective than *M. pulcherrima* MACH1 and GS9 in the control of blue mold rot which results in a stronger reduction in PAT on four cultivar of apples (Spadaro et al., 2013). Thus, it can be speculated that in the samples of plant raw materials being a mixture of various plant parts, competition between species of mold and yeast for living space and nutrients may result in the presence of mold species responsible for the production or inhibition of PAT biosynthesis. However, further research is needed to clarify this hypothesis. In additional, PAT may act synergistically with other mycotoxins such as citrinin, causing more extensive effect on human tissues and organs (Qin et al., 2020). The presence of mycotoxins other than PAT in food is also a serious health problem (Moretti et al., 2017).

## 4 CONCLUSIONS

The conducted research indicates the possibility of using the ELISA method for the determination of PAT in materials of plant origin. Very restrictive technological procedures, such as composition of herbal blends, fruit selection, the conditions of harvesting, selecting, storing, drying the fruits, and distributing it, can significantly reduce the concentration of PAT in dried herbal blends. The search for such eco‐friendly technological method creating the most unfavorable conditions for the biosynthesis of PAT in medicinal raw materials is extremely important from the point of view of medical and pharmaceutical care. Moreover, studies have shown that the percentages of individual components in herbal blends can play a role in the final concentration of PAT in the raw materials. However, these studies are pilot studies and require further improvement. In summary, the results of the research indicate an urgent need to control the degree of contamination of plant materials, dietary supplements, and herbal blends for their PAT content.

## CONFLICT OF INTEREST

No potential conflict of interest was reported by the authors.

## AUTHOR CONTRIBUTIONS

**Anna Przybylska:** Conceptualization (lead); Data curation (lead); Methodology (equal); Software (equal); Visualization (lead); Writing‐original draft (lead); Writing‐review & editing (lead). **Agnieszka Chrustek:** Data curation (supporting); Formal analysis (supporting); Methodology (equal); Software (supporting); Writing‐review & editing (supporting). **Dorota Olszewska‐Słonina:** Formal analysis (equal); Writing‐review & editing (supporting). **Marcin Koba:** Formal analysis (equal); Funding acquisition (lead); Writing‐review & editing (supporting). **Stefan Kruszewski:** Funding acquisition (lead); Project administration (equal).

## ETHICAL APPROVAL

This study does not involve any human or animal testing.

## Data Availability

All data obtained during the research appear in the submitted article.

## References

[fsn32386-bib-0051] Azam, M. S., Ahmed, S., Islam, M. N., Maitra, P., Islam, M. M., Yu, D. (2021). Critical assessment of mycotoxins in beverages and their control measures. Toxins, 13(5), 323. 10.3390/toxins13050323 33946240 PMC8145492

[fsn32386-bib-0001] Amoah, R. E., Kalakandan, S., Wireko‐Manu, F. D., Oduro, I., Saalia, F. K., & Owusu, E. (2020). The effect of vinegar and drying (Solar and Open Sun) on the microbiological quality of ginger (ZINGIBER OFFICINALE ROSCOE) rhizomes. Food Science and Nutrition, 8, 6112–6119. 10.1002/fsn3.1902 33282262 PMC7684629

[fsn32386-bib-0002] Asl Roosta, R., Moghaddasi, R., & Hosseini, S. S. (2017). Export target markets of medicinal and aromatic plants. Journal of Applied Research on Medicinal and Aromatic Plants, 7, 84–88. 10.1016/j.jarmap.2017.06.003

[fsn32386-bib-0003] Barad, S., Sionov, E., & Prusky, D. (2016). Role of patulin in post‐harvest diseases. Fungal Biology Reviews, 30(1), 24–32. 10.1016/j.fbr.2016.02.001

[fsn32386-bib-0004] Do, K. H., An, T. J., Oh, S.‐K., & Moon, Y. (2015). Nation‐based occurence and endogenous biological reduction of mycotoxins in medicinal herbs and species. Toxins, 7, 4111–4130. 10.3390/toxins7104111 26473926 PMC4626724

[fsn32386-bib-0005] Drusch, S., & Ragab, W. (2003). Mycotoxins in fruits, fruit juices, and dried fruits. Journal of Food Protection, 66(8), 1514–1527. 10.4315/0362-028X-66.8.1514 12929850

[fsn32386-bib-0006] Dungkokkruad, P., Jutirak, J., Wongkamsom, N., Kongsee, P., Saichi, M., & Meesa‐ad, A. (2017). The ability of thai herbal household plant crude extracts (alpinia galangal) in growth inhibition of mold aspergillus flavus and destruction of aflatoxin B1. European Journal of Sustainable Development, 6(2), 210. 10.14207/ejsd.2017.v6n2p210

[fsn32386-bib-0007] Fritsche, J. (2018). Recent developments and digital perspectives in food safety and authenticity. Journal of Agricultural and Food Chemistry, 66(29), 7562–7567. 10.1021/acs.jafc.8b00843 29920081

[fsn32386-bib-0008] Hemmami, H., Ben Seghir, B., Ben Ali, M., Rebiai, A., Zeghoud, S., & Brahmia, F. (2020). Phenolic profile and antioxidant activity of bee pollen extracts from different regions of Algeria. Ovidius University Annals of Chemistry, 2(31), 93–98. 10.2478/auoc-2020-0017

[fsn32386-bib-0009] Hussain, S., Asi, M. R., Iqbal, M., Khalid, N., Wajih‐Ul‐Hassan, S., & Ariño, A. (2020). Patulin mycotoxin in mango and orange fruits, juices, pulps, and jams marketed in Pakistan. Toxins, 12(1), 52. 10.3390/toxins12010052 PMC7020514 31963183

[fsn32386-bib-0010] Ioi, J. D., Zhou, T., Tsao, R., & Marcone, M. F. (2017). Mitigation of Patulin in fresh and processed foods and beverages. Toxins, 9(5), 157. 10.3390/toxins9050157 PMC5450705 28492465

[fsn32386-bib-0011] Ji, X., Li, R., Yang, H., Qi, P., Xiao, Y., & Qian, M. (2017). Occurrence of patulin in various fruit products and dietary exposure assessment for consumers in China. Food Control, 78, 100–107. 10.1016/j.foodcont.2017.02.044

[fsn32386-bib-0012] Jin, L., Cai, Y., Sun, C., Huang, Y., & Yu, T. (2019). Exogenous L‐glutamate treatment could induce resistance against *Penicillium expansum* in pear fruit by activating defense‐related proteins and amino acids metabolism. Postharvest Biology and Technology, 150, 148–157. 10.1016/j.postharvbio.2018.11.009

[fsn32386-bib-0013] Kendall, H., Naughton, P., Kuznesof, S., Raley, M., Dean, M., Clark, B., Stolz, H., Home, R., Chan, M. Y., Zhong, Q., Brereton, P., & Frewer, L. J. (2018). Food fraud and the perceived integrity of European food imports into China. PLoS One, 13(5), e0195817. 10.1371/journal.pone.0195817 29791434 PMC5965827

[fsn32386-bib-0014] Kunachowicz, H.; Przygoda, B.; Nadolna, I.; Iwanow, K. Food Composition Tables. 2nd Edition. : PZWL; 2018:608–625.

[fsn32386-bib-0015] Lai, T., Sun, Y., Liu, Y., Li, R., Chen, Y., & Zhou, T. (2021). Cinnamon oil inhibits *Penicillium expansum* growth by disturbing the carbohydrate metabolic process. Journal of Fungi, 7(2), 123. 10.3390/jof7020123 33572180 PMC7915993

[fsn32386-bib-0016] Li, F., Zhao, S., Chin, L., Li, Y., Wu, D., Zhao, X., Han, C., Zhang, H., & Ji, R. (2007). Determination of patulin in apple and Hawthorn beverages by solid‐phase filtration column and liquid chromatography. Journal of AOAC International, 26, 2135. 10.1093/jaoac/90.1.167 17373448

[fsn32386-bib-0017] Lien, K. W., Ling, M. P., & Pan, M. H. (2020). Probabilistic risk assessment of patulin in imported apple juice and apple‐containing beverages in Taiwan. Journal of the Science of Food and Agriculture, 100, 4776–4781. 10.1002/jsfa.10536 32458424

[fsn32386-bib-0018] McCoy, S., Chang, J. W., McNamara, K. T., Oliver, H. F., & Deering, A. J. (2015). Quality and safety attributes of afghan raisins before and after processing. Food Science and Nutrition, 3(1), 56–64. 10.1002/fsn3.190 25650241 PMC4304563

[fsn32386-bib-0019] Montaseri, H., Eskandari, M. H., Yeganeh, A. T., Karami, S., Javidnia, K., Dehghanzadeh, G. R., Mesbahi, G. R., & Niakousari, M. (2013). Patulin in apple leather in Iran. Food Additives and Contaminants: Part B, 7(2), 2014. 10.1080/19393210.2013.855825 24914594

[fsn32386-bib-0020] Moretti, A., Logrieco, A. F., & Susca, A. (2017). Mycotoxins: An underhand food problem. In A. F. Logrieco (Eds.), Methods in molecular biology (1542 edn., pp. 3–12). 10.1007/978-1-4939-6707-0_1 27924528

[fsn32386-bib-0021] Ogrodowska, D., Zadernowski, R., Tańska, M., & Czaplicki, S. (2011). Physical properties of *Amaranthus cruentus* seeds from different cultivation regions in Poland. Food Science Technology Quality, 6(79), 91–104.

[fsn32386-bib-0052] Omar S. S., Haddad M. A., Parisi S. (2020). Validation of HPLC and Enzyme‐Linked Immunosorbent Assay (ELISA) techniques for detection and quantification of Aflatoxins in different food samples. Foods, 9(5), 661. 10.3390/foods9050661 PMC7278759 32443841

[fsn32386-bib-0022] Orina, A. S., Gavrilova, O. P., Gagkaeva, T. Y., Burkin, A. A., & Kononenko, G. P. (2020). The contamination of Fabaceae plants with fungi and mycotoxins. Agricultural and Food Science, 29(3), 265–275. 10.23986/afsci.89171

[fsn32386-bib-0023] Pal, S., Singh, N., & Ansari, K. M. (2017). Toxicological effects of patulin mycotoxin on the mammalian system: An overview. Toxicology Research, 6(6), 764–771. 10.1039/c7tx00138j 30090541 PMC6062217

[fsn32386-bib-0024] Pobiega, K., Kraśniewska, K., & Gniewosz, M. (2019). Application of propolis in antimicrobial and antioxidative protection of food quality – A review. Trends in Food Science and Technology, 83, 53–62. 10.1016/j.tifs.2018.11.007

[fsn32386-bib-0025] Popa, M. E., Catana, L., Popa, E. E., Mitelut, A. C., Tylewicz, U., & Dalla Rosa, M. (2019). Patulin analysis of some organic dried fruits samples by HPLC‐DAD. Romanian Biotechnological Letters, 24, 1–9. 10.25083/rbl/24.3/491.498

[fsn32386-bib-0026] Przybylska, A., Bazylak, G., Kosicki, R., Ałtyn, I., Twarużek, M., & Grajewski, J. (2018). The content of patulin in dietary supplements and herbal blends comprising of dried hawthorn fruit. Bromatology and Toxicological Chemistry, 51(3), 161–168.

[fsn32386-bib-0027] Przybylska, A., Bazylak, G., Kosicki, R., Altyn, I., Twarużek, M., Grajewski, J., & Sołtys‐Lelek, A. (2019). Advantageous extraction, cleanup, and UHPLC‐MS/MS detection of patulin mycotoxin in dietary supplements and herbal blends containing hawberry from *Crataegus* spp. Journal of Analytical Methods in Chemistry. 2019, ID 2159097, 13. 10.1155/2019/2159097 PMC6381574 30881725

[fsn32386-bib-0028] Przybylska, A., Gackowski, M., & Koba, M. (2021). Application of capillary electrophoresis to the analysis of bioactive compounds in herbal raw materials. Molecules, 26, 2135. 10.3390/molecules26082135 33917716 PMC8068163

[fsn32386-bib-0029] Puel, O., Galtier, P., & Oswald, I. P. (2010). Biosynthesis and toxicological effects of patulin. Toxins, 2(4), 613–631. 10.3390/toxins2040613 22069602 PMC3153204

[fsn32386-bib-0030] Puga‐Torres, B., Salazar, D., Cachiguango, M., Cisneros, G., & Gómez‐Bravo, C. (2020). Determination of aflatoxin M1 in raw milk from different provinces of Ecuador. Toxins, 12(8), 498. 10.3390/toxins12080498 PMC7472276 32756414

[fsn32386-bib-0031] Qin, L., Jiang, J. Y., Zhang, L., Dou, X. W., Ouyang, Z., Wan, L., & Yang, M. H. (2020). Occurrence and analysis of mycotoxins in domestic Chinese herbal medicines. Mycology, 11, 146. 10.1080/21501203.2020.1727578 PMC7448902 32923021

[fsn32386-bib-0032] Reinholds, I., Bogdanova, E., Pugajeva, I., Alksne, L., Stalberga, D., Valcina, O., & Bartkevics, V. (2020). Determination of fungi and multi‐class mycotoxins in *Camelia sinensis* and herbal teas and dietary exposure assessment. Toxins, 12(9), 555. 10.3390/toxins12090555 PMC7551389 32872457

[fsn32386-bib-0033] Reinholds, I., Bogdanova, E., Pugajeva, I., & Bartkevics, V. (2019). Mycotoxins in herbal teas marketed in Latvia and dietary exposure assessment. Food Additives and Contaminants: Part B Surveillance, 12(3), 199–208. 10.1080/19393210.2019.1597927 30961455

[fsn32386-bib-0034] Sergunova, E. V., Bokov, D. O., Bokov, D. O., Bobkova, N. V., Kovaleva, T. Y., Chromchenkova, E. P., & Bessonov, V. V. (2020). Amino acid profile and content in crude herbal drugs (fruits) of Rosaceae species. Systematic Reviews in Pharmacy, 11(5), 322–329. 10.31838/srp.2020.5.47

[fsn32386-bib-0035] Silici, S., & Karaman, K. (2014). Inhibitory effect of propolis on patulin production of penicillium expansum in apple juice. Journal of Food Processing and Preservation, 38(3), 1129–1134. 10.1111/jfpp.12072

[fsn32386-bib-0036] Singh, J., & Mehta, A. (2020). Rapid and sensitive detection of mycotoxins by advanced and emerging analytical methods: A review. Food Science and Nutrition, 8(5), 2183–2204. 10.1002/fsn3.1474 32405376 PMC7215233

[fsn32386-bib-0037] Sohrabi, H., Arbabzadeh, O., Khaaki, P., Khataee, A., Majidi, M. R., & Orooji, Y. (2021). Patulin and Trichothecene: Characteristics, occurrence, toxic effects and detection capabilities via clinical, analytical and nanostructured electrochemical sensing/biosensing assays in foodstuffs. Critical Reviews in Food Science and Nutrition, 10.1080/10408398.2021.1887077 33624529

[fsn32386-bib-0038] Spadaro, D., Lorè, A., Garibaldi, A., & Gullino, M. L. (2013). A new strain of *Metschnikowia fructicola* for postharvest control of *Penicillium expansum* and patulin accumulation on four cultivars of apple. Postharvest Biology and Technology, 75, 1–8. 10.1016/j.postharvbio.2012.08.001

[fsn32386-bib-0039] Sudakin, D., & Fallah, P. (2008). Toxigenic fungi and mycotoxins in outdoor, recreational environments. Clinical Toxicology, 46(8), 738–744. 10.1080/15563650701687443 18615277

[fsn32386-bib-0040] Tang, E. N., Ndindeng, S. A., Bigoga, J., Traore, K., Silue, D., & Futakuchi, K. (2019). Mycotoxin concentrations in rice from three climatic locations in Africa as affected by grain quality, production site, and storage duration. Food Science and Nutrition, 7(4), 1274–1287. 10.1002/fsn3.959 31024700 PMC6475755

[fsn32386-bib-0041] Tangni, E. K., & Pussemier, L. (2007). Ergosterol and mycotoxins in grain dusts from fourteen Belgian cereal storages: A preliminary screening survey. Journal of the Science of Food and Agriculture, 87(7), 1263–1270. 10.1002/jsfa.2838

[fsn32386-bib-0042] Tannous, J., Atoui, A., El Khoury, A., Francis, Z., Oswald, I. P., Puel, O., & Lteif, R. (2016). A study on the physicochemical parameters for *Penicillium expansum* growth and patulin production: Effect of temperature, pH, and water activity. Food Science and Nutrition, 4(4), 611–622. 10.1002/fsn3.324 27386110 PMC4930504

[fsn32386-bib-0043] Thirumala‐Devi, K., Mayo, M. A., Reddy, G., Emmanuel, K. E., Larondelle, Y., & Reddy, D. V. R. (2001). Occurrence of ochratoxin A in black pepper, coriander, ginger and turmeric in India. Food Additives and Contaminants, 18(9), 830–835. 10.1080/02652030117589 11552750

[fsn32386-bib-0044] Tonti, S., Mandrioli, M., Nipoti, P., Pisi, A., Toschi, T. G., & Prodi, A. (2017). Detection of fumonisins in fresh and dehydrated commercial garlic. Journal of Agricultural and Food Chemistry, 65(32), 7000–7005. 10.1021/acs.jafc.7b02758 28719747

[fsn32386-bib-0045] Vaclavikova, M., Dzuman, Z., Lacina, O., Fenclova, M., Veprikova, Z., Zachariasova, M., & Hajslova, J. (2015). Monitoring survey of patulin in a variety of fruit‐based products using a sensitive UHPLC‐MS/MS analytical procedure. Food Control, 47, 577–584. 10.1016/j.foodcont.2014.07.064

[fsn32386-bib-0046] Xiang, L., Gao, Y. H., Liu, D. Y., & Yang, M. H. (2012). Rapid determination of patulin in medicinal hawthorn fruits by HPLC‐MS/MS. World Mycotoxin Journal, 5(1), 31–36. 10.3920/WMJ2011.1336

[fsn32386-bib-0047] Zenkova, M., & Pinchykova, J. (2019). Chemical composition of sea‐buckthorn and highbush blueberry fruits grown in the Republic of Belarus. Food Science and Applied Biotechnology, 2(2), 121–129. 10.30721/fsab2019.v2.i2.59

[fsn32386-bib-0048] Zhang, L., Dou, X. W., Zhang, C., Logrieco, A. F., & Yang, M. H. (2018). A review of current methods for analysis of mycotoxins in herbal medicines. Toxins, 10(2), 65. 10.3390/toxins10020065 PMC5848166 29393905

[fsn32386-bib-0049] Zhang, Y., Skaar, I., Sulyok, M., Liu, X., Rao, M., & Taylor, J. W. (2016). The microbiome and metabolites in fermented Pu‐erh tea as revealed by high‐throughput sequencing and quantitative multiplex metabolite analysis. PLoS One, 11(6), e0157847– 10.1371/journal.pone.0157847 27337135 PMC4918958

[fsn32386-bib-0050] Zhou, Y., Kong, W., Li, Y., Logrieco, A. F., Xu, J., & Yang, M. (2012). A new solid‐phase extraction and HPLC method for determination of patulin in apple products and hawthorn juice in China. Journal of Separation Science, 35(5–6), 641–649. 10.1002/jssc.201100919 22517639

